# Enhanced Impact Properties of Hybrid Composites Reinforced by Carbon Fiber and Polyimide Fiber

**DOI:** 10.3390/polym13162599

**Published:** 2021-08-05

**Authors:** Boyao Wang, Bin He, Zhanwen Wang, Shengli Qi, Daijun Zhang, Guofeng Tian, Dezhen Wu

**Affiliations:** 1 State Key Laboratory of Chemical Resource Engineering, Beijing University of Chemical Technology, Beijing 100029, China; 2018210146@buct.edu.cn (B.W.); hebin90s@163.com (B.H.); 13011142122@163.com (Z.W.); qisl@mail.buct.edu.cn (S.Q.); 2 National Key Laboratory of Advanced Composite Materials, Aero Engine Corporation of China Beijing Institute of Aeronautical Materials, Beijing 100095, China; 15810534483@139.com

**Keywords:** polymer-matrix composites (PMCs), hybrid, Charpy impact strength, polyimide fiber

## Abstract

A series of hybrid fiber-reinforced composites were prepared with polyimide fiber and carbon fiber as the reinforcement and epoxy resin as the matrix. The influence of stacking sequence on the Charpy impact and flexural properties of the composites as well as the failure modes were studied. The results showed that hybrid fiber-reinforced composites yielded nearly 50% increment in Charpy impact strength compared with the ones reinforced by carbon fiber. The flexural performance was significantly improved compared with those reinforced solely by polyimide fibers and was greatly affected by the stacking sequence. The specimens with compressive sides distributed with carbon fiber possessed higher flexural strength, while those holding a sandwich-like structure with carbon fiber filling between the outer layers displayed a higher flexural modulus.

## 1. Introduction

A typical representative of advanced composites, resin-based composites reinforced by carbon fiber with features such as being light weight and having high strength and modulus have found broad applications in the fields of upmarket sports equipment and aerospace equipment [1,2,3]. However, the high rigidity of carbon fiber incurs brittleness and poor toughness [4,5,6,7], which makes the composites prepared show relatively low impact resistance. Hybrid-fiber reinforcement can take full advantage of the performance superiorities of two or more kinds of fibers to make up for the deficiencies of a single reinforcement pattern in certain properties [8,9,10]. Mixing carbon fiber with ones possessing high toughness and ductility has been proposed as another effective way to improve the toughness of original composites [11,12].

Polyimide (PI) fiber with high strength and high modulus is a kind of high-performance organic fiber that has newly emerged in recent years. It has excellent mechanical properties, tolerance to high and low temperatures, environmental resistance, little moisture absorption, low dielectric, and high insulation [13,14,15,16,17,18,19,20,21]. PI fiber is composed of amorphous polymer with highly oriented molecular chains. When impacted by external force, it can absorb and dissipate energy through chain movement; the timely conversion of kinetic energy into thermal energy required by the thermal motion of molecular chains endows the fiber with excellent toughness. Our previous research indicated PI fiber underwent plastic deformation during quasi-static tension, while fibrillation occurred during high-strain-rate tension, both of which were conducive to energy absorption and thereby toughness enhancement [22]. Therefore, we decided to explore whether PI fibers and carbon fibers could be simultaneously adopted to prepare hybrid fiber-reinforced composites (HFRP), so as to make full use of the high toughness of PI fiber to balance the stiffness and toughness of carbon fiber-reinforced composites (CFRP), and in the meantime, improve the impact resistance of composites. In addition, PI fiber has a density as low as 1.44 g/cm^3^, which is about 20% lower than that of carbon fiber, so it could also play a role in structural weight reduction. HFRP is expected to be used as an impact-resistant material in automotive, aerospace, and other fields.

Herein, with S35 PI fiber fabric and T700 carbon fiber fabric used as reinforcements and epoxy resin as the matrix, a series of HFRP laminates were prepared by solution-based prepreg combined via the hot molding method. The effects of stacking sequence on the impact and flexural properties of the composites were systematically studied, and the failure mode of the specimens was observed and analyzed in detail. It is expected that our research can provide new design ideas for stacking sequence and material selection schemes for impact-resistant composites [23,24,25].

## 2. Experiments

### 2.1. Materials

Carbon fiber fabric (T700-12K) with an areal density of 200 g/m^2^ was commercially available. The PI fiber (S35, Jiangsu Shino New Materials & Technology Co., Ltd, Changzhou, China) fabric had an areal density of 200 g/m^2^. Epoxy resin (4,5-epoxycyclohexane-1,2-dicarboxylic acid diglycidyl ester, TDE-85) was obtained from Tianjin Jingdong Chemical Composites Co., Ltd, Tianjin, China. The curing agent (3,3′-diethyl-4,4′-diaminodiphenylmethane, DEDDM) was produced by Jiangyin Huifeng Electronic Materials Co., Ltd, Wuxi, China. The acetone was produced by Beijing Chemical Plant, Beijing, China. Mechanical parameters of the raw fibers are listed in Table 1.

### 2.2. Preparation

**Scheme 1:**
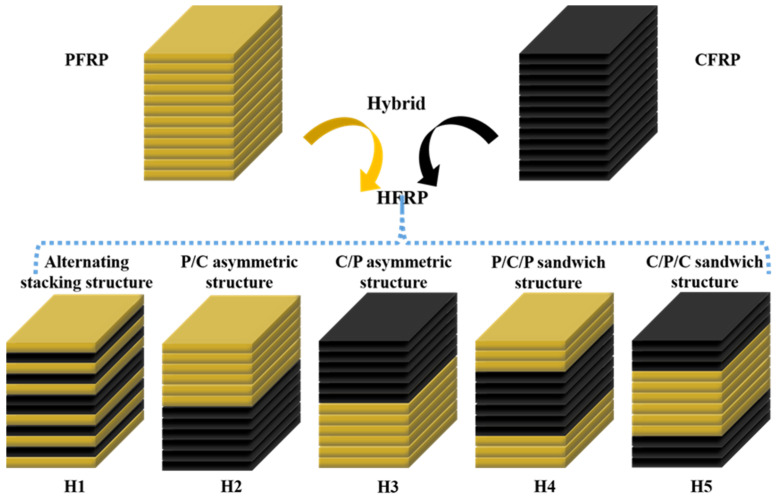
Stacking sequence of HFRP.

TDE-85 and DEDDM were mixed at a mass ratio of 0.65:0.35, and then added to an appropriate amount of acetone to prepare the resin solution. The fabric was cut into the required sizes to fabricate the prepreg through a solution-based method. After hot-press curing, a series of fabric-reinforced composite laminates were obtained. The curing procedure was 125 °C/2 h + 165 °C/3 h + 185 °C/3 h, which was in accordance with previous work [26], and the curing pressure was 3 MPa. In this work, the hybrid ratio was defined as the mass percentage of carbon fiber in the total fiber. The specific fabrication details under each laminate code are described in detail in Table 2 and Scheme 1. H1 stands for the interlaminar hybrid of alternating stacking structure. H2 and H3 represent asymmetric stacking structure. H4 and H5 have sandwich-like structures, with the outer layers being PI fiber and carbon fiber, respectively.

### 2.3. Characterization

The mechanical testing standards for composite laminates in this work are detailed in Table 3. A HIT 25P pendulum impact testing machine was used to evaluate Charpy impact properties. The impact was directed vertically through the layers, and the span-to-depth ratio was 20. Flexural properties were examined on an Instron 5966 electronic universal material testing machine. Three-point bending was applied for the bending test. The span-to-depth ratio was 32 for the bending specimens. There were five specimens in each group, and the average values were reported. The macroscopic appearance was recorded with a digital camera and an optical microscope.

## 3. Results and Discussion

### 3.1. Charpy Impact Properties

**Figure 1:**
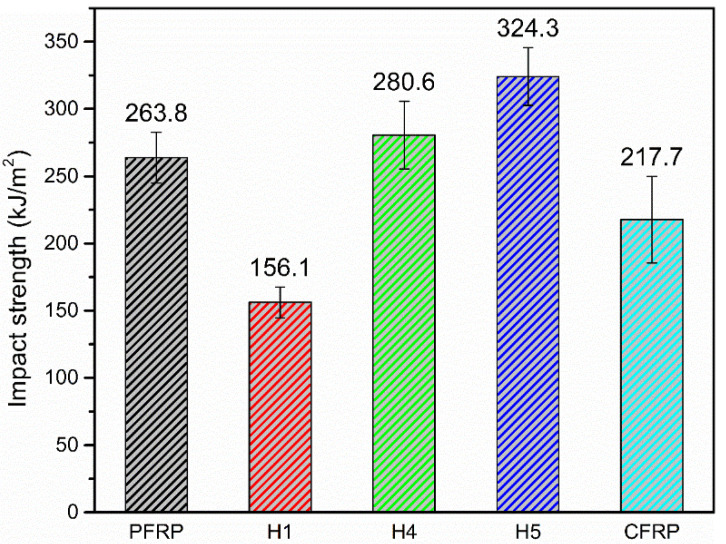
Impact strength of the composites.

Charpy impact strengths of the composites are summarized in Figure 1, which reveals an order of H1 < CFRP < PFRP < H4 < H5. The error bars represent standard deviation. PFRP demonstrated a higher impact strength than that of CFRP (263.8 kJ/m^2^ versus 217.2 kJ/m^2^), indicative of its better impact resistance. The impact strength of HFRP was strongly related to the stacking sequence. Specifically, the sandwich structure was more conducive to the improvement of impact strength, with 280.6 kJ/m^2^ and 324.3 kJ/m^2^ obtained by H4 and H5, respectively, which are much higher values than those of PFRP and CFRP. H5 achieved the highest impact strength and an increment of approximately 50% compared with CFRP, exhibiting better impact resistance. The remarkable enhancement for H5 might be explained as follows: the carbon fiber outer layer was beneficial to the rapid diffusion of stress waves because of its high modulus, and PI fiber in the core layer was favorable for the absorption of impact energy due to its high elongation at breaking point.

**Figure 2:**
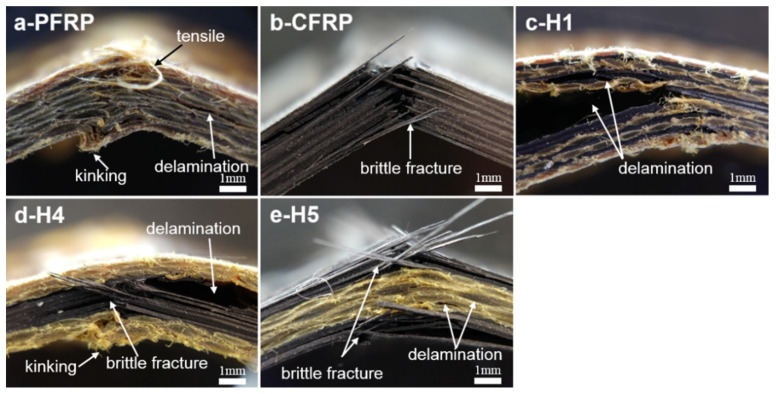
Morphology of surfaces of composite specimens after impact test: (**a**) PFRP, (**b**) CFRP, (**c**) H1, (**d**) H4, and (**e**) H5.

The morphology of the surfaces of the composites after the Charpy impact test was further characterized by optical microscopy (Figure 2). Delamination was found in the PFRP specimen; the PI fiber layer on the compressive side yielded and showed kinking failure, while that on the tensile side underwent tensile failure with some fiber broken and pulled out (Figure 2a). On the other hand, overall fracture with a through-thickness break could be clearly observed for CFRP, and the broken fiber presented sharp brittle fractures (Figure 2b).

Figure 2c–e suggest that none of the HFRPs underwent complete fracture, and all the specimens maintained good integrity. Delamination occurred in all three cases, although the delamination situation was different in each case. It took place at the interface of PI fiber/carbon fiber in the alternating stacking structure (H1). For the sandwich-like H4, the inner carbon fiber layer suffered brittle fracture; meanwhile, delamination occurred at the PI fiber/carbon fiber interface on the tensile side, and kinking failure appeared on the compressive side. H5 also held a sandwich structure but in an inverse pattern; the carbon fiber outer layer failed on both the tensile side and compressive side similarly to the CFRP specimen, while multiple delamination occurred in the inner PI fiber layer.

### 3.2. Flexural Properties

**Figure 3:**
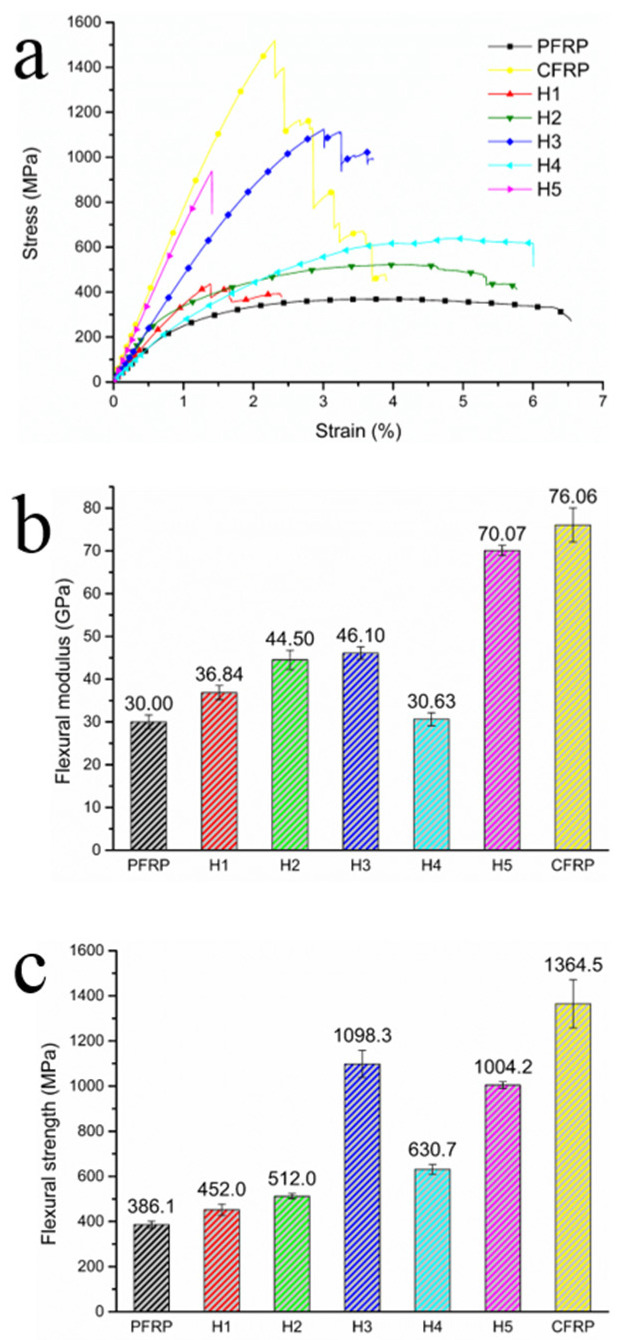
Flexural properties of the composites: (**a**) flexural stress–strain curves, (**b**) flexural modulus, and (**c**) flexural strength.

Flexural stress–strain curves of the composite specimens are plotted in Figure 3a. The curves represent the average value of the samples. The stiffness of carbon fibers imparted a good linearity to the CFRP curve, which rose steeply at first and dropped instantly upon reaching the critical stress, indicative of high flexural strength, high flexural modulus, and low failure strain of CFRP. As for PFRP, the ductility of PI fibers resulted in a nonlinear curve. Therefore, PFRP possessed low flexural strength, low flexural modulus, but a failure strain over 6.5%, which is much higher than that of CFRP and thus shows excellent toughness. 

The flexural modulus of PFRP and CFRP was 30.0 and 76.1 GPa, respectively, while that of HFRP (H1–H5) was in between these values (Figure 3b). The order of flexural modulus of HFRP followed H5 > H3 > H2 > H1 > H4. The partial replacement of PI fibers with carbon fibers could enhance the flexural modulus to various degrees as the layer sequence varied. With the outer layer consisting of carbon fiber, the sandwich-like H5 showed the highest flexural modulus of 70.1 GPa, which reached 92% of that of CFRP.

The flexural strengths of the composites are compared in Figure 3c. The flexural strength of PFRP and CFRP was 386.1 and 1364.5 MPa, respectively, and that of HFRP (H1–H5) fell in between these values. The introduction of carbon fiber caused a marked improvement, which also largely depended on the stacking sequence. Carbon fiber being in the outer layer (i.e., H5) was more beneficial than being in the core layer (i.e., H4). Replacing PI fiber with carbon fiber in the outer layer of the compressive side to withstand more compressive stress could significantly improve the flexural strength of HFRP. Among all the HFRP specimens, H5 maintained excellent flexural strength of approximately 1004 MPa, which was 2.6 times that of PFRP and 75% of CFRP.

**Figure 4:**
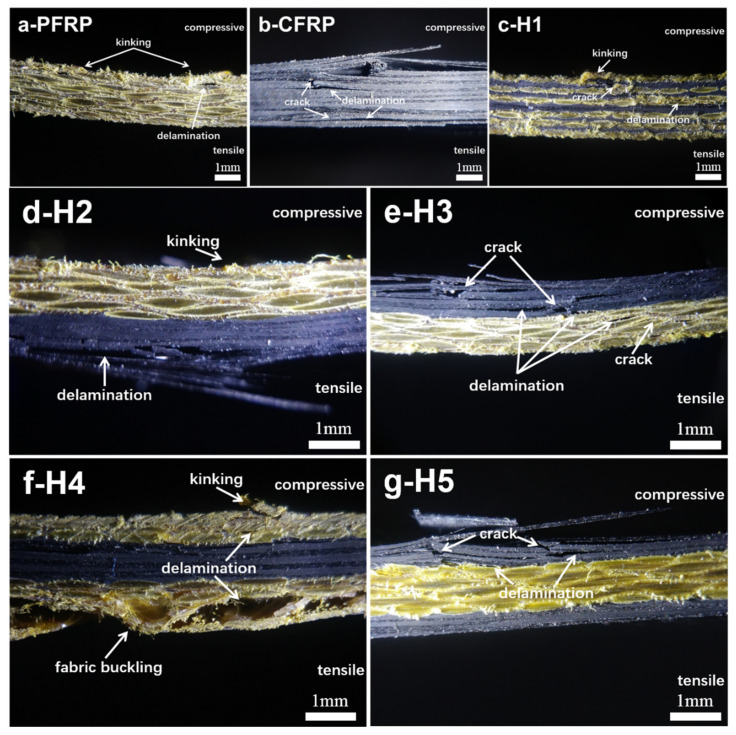
Morphology of the surface of failed composite laminates under flexural loading: (**a**) PFRP, (**b**) CFRP, (**c**) H1, (**d**) H2, (**e**) H3, (**f**) H4, (**g**) H5.

According to the morphology of the surface in Figure 4, PFRP showed kinking failure on the compressive side but no fracture, which can be attributed to the toughness of PI fiber. CFRP underwent both tensile and compressive failure, and crack propagation led to multiple delamination sites on the compressive side. The compressive side of H2 had no crack propagation due to the toughness of PI fiber, for which H2 had a large flexural failure strain. As for H3, cracks on the compressive side propagated from the carbon fiber layer to the inner PI fiber layer, triggering delamination similar to CFRP. With both outer layers being PI fiber, H4 showed large deformations on the compressive side along with obvious failure on the tensile side, demonstrated by delamination and fabric buckling. Cracks occurred in the carbon fiber layer on the compressive side of H5 and propagated throughout the entire layer, but it could not pass through the PI fiber layer. In general, the continuous carbon fiber layer was prone to crack propagation, while the PI fiber layer could block crack propagation due to its excellent toughness. When set in a suitable position, the PI fiber layer could afford greater flexural strain. By delicate design of the stacking sequences of carbon fiber and PI fiber, HFRP with good flexural properties that can meet the needs of practical applications can be obtained.

## 4. Conclusions

PI fiber fabric and carbon fiber fabric were used as reinforcements and epoxy resin was used as the matrix to prepare a series of interlayer hybrid composites. The effects of stacking sequence on the basic mechanical properties and failure modes of the composites have been discussed. Experimental results showed that stacking sequence could make a difference to the Charpy impact properties and flexural properties of HFRP. The Charpy impact strength of sandwich-like structures with the outer layers being carbon fiber (H5) was nearly 50% higher than that of CFRP, and its flexural strength also maintained 75% of that of CFRP, which demonstrates excellent structural strength. Further, carbon fiber on the compressive side was very helpful for the improvement of flexural strength and modulus.

In conclusion, with reasonable structural design, sandwich-like structures could improve the impact resistance of hybrid composites to a large extent, and maintain the flexural performance of the hybrid composites.

## Figures and Tables

**Table 1:** Constituent materials and selected properties.

Material	Tensile Strength (MPa)	Tensile Modulus (GPa)	Elongation (%)	Density (g/cm^3^)
Carbon fiber (T700-12K)	4900	230	2.1	1.80
PI fiber (S35)	3500	120	>3.0	1.44

**Table 2:** Stacking sequence of hybrid composites.

Laminate Code	Ply Number Ratio (Carbon/Polyimide)	Stacking Sequence	Hybrid Ratio (%)
PFRP	0/12	⚪⚪⚪⚪⚪⚪⚪⚪⚪⚪⚪⚪	0
CFRP	12/0	⚫⚫⚫⚫⚫⚫⚫⚫⚫⚫⚫⚫	100
H1	6/6	⚪⚫⚪⚫⚪⚫⚫⚪⚫⚪⚫⚪	50
H2	6/6	⚪⚪⚪⚪⚪⚪⚫⚫⚫⚫⚫⚫	50
H3	6/6	⚫⚫⚫⚫⚫⚫⚪⚪⚪⚪⚪⚪	50
H4	6/6	⚪⚪⚪⚫⚫⚫⚫⚫⚫⚪⚪⚪	50
H5	6/6	⚫⚫⚫⚪⚪⚪⚪⚪⚪⚫⚫⚫	50

Note: ⚪ represents polyimide fiber, ⚫ represents carbon fiber.

**Table 3:** Mechanical property measurements.

Tests	Standard	Specimen Dimensions (mm × mm × mm)	Loading Rate (mm/min)
Impact test	GB/T 1043.1-2008	75 × 10 × 2	/
Three-point bending test	ASTM D7264	80 × 13 × 2	2

## Data Availability

The data presented in this study are available on request from the corresponding author.
